# Associations of recent weight loss with health care costs and utilization among older women

**DOI:** 10.1371/journal.pone.0191642

**Published:** 2018-01-29

**Authors:** John T. Schousboe, Allyson M. Kats, Lisa Langsetmo, Brent C. Taylor, Tien N. Vo, Deborah M. Kado, Howard A. Fink, Kristine E. Ensrud

**Affiliations:** 1 HealthPartners Institute, HealthPartners, Minneapolis, Minnesota, United States of America; 2 Division of Health Policy and Management, University of Minnesota, Minneapolis, Minnesota, United States of America; 3 Division of Epidemiology and Community Health, University of Minnesota, Minneapolis, Minnesota, United States of America; 4 Department of Medicine, University of Minnesota, Minneapolis, Minnesota, United States of America; 5 Center for Chronic Disease Outcomes Research, Minneapolis VA Health Care System, Minneapolis, Minnesota, United States of America; 6 University of California, San Diego, California, United States of America; 7 Geriatric Research Education and Clinical Center, Minneapolis VA Health Care System, Minneapolis, Minnesota, United States of America; Medical University of Vienna, AUSTRIA

## Abstract

The association of weight loss with health care costs among older women is uncertain. Our study aim was to examine the association of objectively measured weight change with subsequent total health care (THC) costs and other health care utilization among older women. Our study population included 2,083 women (mean age 80.2 years) enrolled in the Study of Osteoporotic Fractures and U.S. Medicare Fee for Service. Weight loss and gain were defined, respectively, as ≥5% decrease and ≥5% increase in body weight, and weight maintenance as <5% change in body weight over a period of 4.5 years. THC costs, outpatient costs, hospitalizations, and skilled nursing facility [SNF] utilization were estimated from Medicare claims for 1 year after the period during which weight change was measured. The associations of weight change with THC and outpatient costs were estimated using generalized linear models with gamma variance and log link functions, and with hospitalizations and SNF utilization using logistic models. Adjusted for age and current body mass index (BMI), weight loss compared with weight maintenance was associated with a 35% increase in THC costs ($2148 [95% CI, 745 to 3552], 2014 U.S. dollars), a 15% increase in outpatient costs ($329 [95% C.I. −1 to 660]), and odds ratios of 1.42 (95% CI, 1.14 to 1.76) for ≥1 hospital stay and 1.45 (95% CI, 1.03 to 2.03) for ≥1 SNF stay. These associations did not vary by BMI category. After additional adjustment for multi-morbidity and functional status, associations of weight loss with all four outcomes were no longer significant. In conclusion, ≥5% weight loss among older women is not associated with increased THC and outpatient costs, hospitalization, and SNF utilization, irrespective of BMI category after accounting for multi-morbidity and impaired functional status that accompany weight loss.

## Introduction

Weight loss in older adults often is associated with incident morbidity and mortality, and hence may be associated with high health care utilization and costs. [1, 2] The relationship between weight loss and costs may be further modified by body mass index (BMI), since high BMI also is associated with increased costs. [3, 4] A study using the Medicare Current Beneficiary Survey data found that weight loss among overweight and obese individuals was associated with increased health care costs among those age 65 to 74, but not among those age 75 and older, though this study was limited in that both BMI and weight-loss were self-reported. [5] No study has focused specifically on the association of *objectively measured* weight change with health care costs among the very old (age 75 or older), and whether this association might differ by BMI. In addition, it is uncertain if an association of weight loss with health care utilization and costs is independent of multimorbidity burden and functional decline,

Using data from the Study of Osteoporotic Fractures linked to Medicare claims among women who were enrolled in Fee for Service, our objective was three-fold; a) to estimate the association of recent weight loss with subsequent total health care costs (as a measure of aggregate health care burden), outpatient costs, hospital stays, and skilled nursing facility (SNF) stays; b) to determine if any association of weight loss with subsequent health care costs and utilization was explained by other characteristics that might be associated with weight loss, specifically multi-morbidity burden, impaired functional status, or poor physical performance; and c) to examine if the association of weight loss with total health care costs varied by BMI category (normal, overweight, or obese).

## Materials and methods

**Fig 1:**
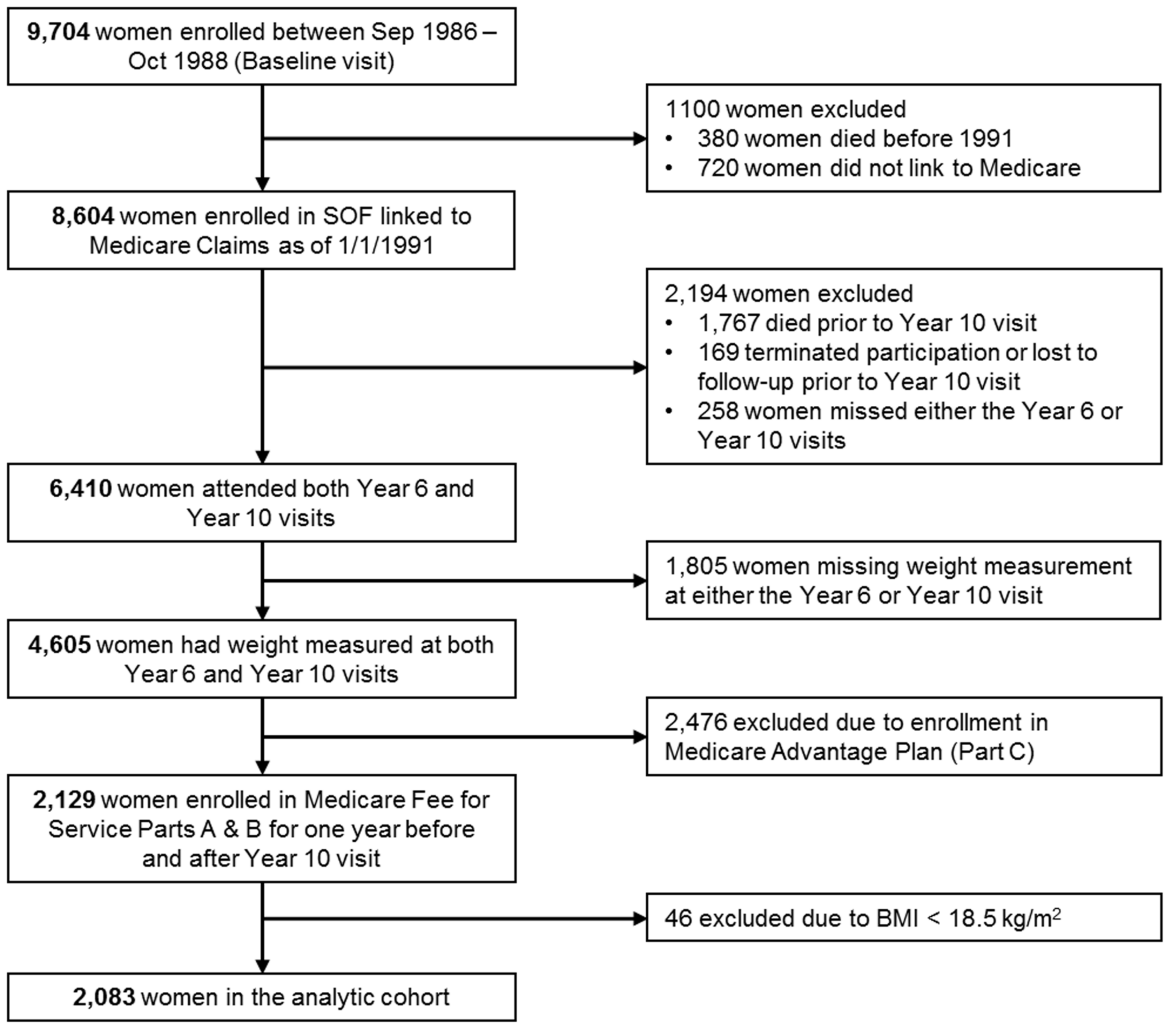
Flow diagram of analysis cohort.

The Study of Osteoporotic Fractures (SOF) recruited 9,704 community-dwelling Caucasian women age 65 or older between 1986 and 1988 from population based listings in four geographic regions of the United States; Baltimore, MD; Minneapolis, MN; Portland, OR; and a rural area (Monongahela Valley) near Pittsburgh, PA. [6] Using previously published validated methods, [7] successful matches to Medicare claims were achieved for 8604 surviving women enrolled in SOF as of January 1, 1991, the earliest date for which outpatient Medicare claims are available (Fig 1).

Women were included in the present analyses if: a) they had weight measured at both the Year 6 SOF study exam between 1992 and 1994 and the Year 10 SOF study exam between 1996 and 1998; and b) were enrolled in Medicare Fee for Service Parts A and B for the 12 months prior to their Year 10 SOF study exam, during the month of their Year 10 SOF study exam, and for the subsequent 12 months or until death, whichever came first (n = 2,129). Women enrolled in Medicare Advantage during any of these time periods were excluded (n = 2,476). Because only 46 women were underweight (BMI<18.5 kg/m^2^), this category was too small for cost analysis, and these individuals were excluded, leaving a study sample size of 2,083 women (Fig 1).

### Participant characteristics

SOF study visits occurred every 2–5 years from 1986 through 2010, with quality control methods described in previous publications. [8] The Year 10 SOF exam was our baseline, and hence our exposure variables were assessed at that exam. Percent weight change, our primary predictor variable, was calculated as the Year 10 exam body weight minus the Year 6 exam body weight, divided by the Year 6 exam body weight. Weight at both visits was measured using a balance beam scale (that was calibrated every two weeks) with the participant in indoor clothing and with shoes removed. The mean time interval between the Year 6 and Year 10 SOF exam dates was 4.5 (SD 0.6) years. Height was measured with a Harpenden stadiometer, and current BMI was calculated as the Year 10 exam weight (kilograms) divided by the height (meters) squared.

At the Year 10 exam, the ability to perform five instrumental activities of daily living (IADLs), [9, 10] health status (recorded as a single question survey item), smoking status, and whether or not the person walked for exercise were assessed by self-report. Grip strength was measured with a hand-held dynamometer. [11] The time required to walk 6 meters at usual pace was measured twice, and these two measures were averaged. We used the 15-item Geriatric Depression Scale (GDS) [12] to measure depressive symptoms and a modification of the Mini-Mental State Examination (mMMSE) [13], scored from 0 to 26, to measure global cognitive function. Our measure of multi-morbidity was based on a modified Elixhauser count [14] enumerating the presence or absence of 31 separate diagnoses from Medicare inpatient and outpatient claims data for the year prior to the date of the Year 10 SOF study exam. Our modified score excluded the diagnoses of obesity, weight loss, and depression since we wished to include very similar variables (weight loss, BMI, and depressive symptoms derived from cohort data) as separate predictors in our analytic models, thereby resulting in a multi-morbidity index ranging from 0 to 28.

### Total health care costs for the year after the SOF year 10 exam

Our primary outcome variable was total health care costs for the 12 months after the SOF Year 10 exam (or until death for the 44 women [2.1%]) who died before the one year anniversary of their Year 10 exam). Total health care costs were calculated as the sum of costs for hospital stays, skilled nursing facility (SNF) stays paid under Medicare part A, inpatient rehabilitation facility (IRF) stays, outpatient care, and home health care for that time period. By incorporating all these components of health care, total health care costs represent a measure of overall health care burden. All hospital stays, Part A paid SNF stays, and IRF stays during that year were identified in the Medical Provider Analysis and Review (MedPAR) file. Standardized costs for hospital stays, SNF stays, and IRF stays were estimated using previously published and validated methods. [7, 15, 16] Costs for Part A paid SNF stays, for IRF stays, home health care utilization, and outpatient utilization were based on the allowable charges for these services in the MedPAR, Home Health Care, Carrier, and Outpatient Medicare claims files. The costs of all units of utilization were adjusted for health care cost inflation to U.S. 2014 dollars, using previously published methods. [7]

### Statistical analysis

Change in body weight between the Year 6 and Year 10 study exams was categorized as weight loss (decrease in body weight of 5% or more), weight gain (increase in body weight of 5% or more), or weight maintenance (weight change less than 5%). BMI at the Year 10 exam was categorized as normal (18.5 to 24.9 kg/m^2^), overweight (25.0 to 29.9 kg/m^2^), or obese (≥30 kg/m^2^). We did not assign those with severe obesity (≥35 kg/m^2^) to a separate category, since only 124 women had BMI at or above this threshold.

Impaired IADLs were categorized as no impairments, one impaired IADL, and two or more impaired IADLs. Multi-morbidity was categorized as none, one or two, three or four, or 5 or more co-morbid conditions. Depressive symptoms were categorized as none or minimal (GDS score of 0 or 1), mild (2 to 5), or moderate to severe (GDS scale score of ≥6). Self-reported health status was dichotomized as fair, poor, or very poor, vs. good or excellent.

We used generalized linear models to estimate the associations of weight change category and other predictors with both total health care and outpatient costs. Based on Modified Park [17] and Pregibon link [18] tests, we chose a log link and gamma distribution for the regression models in order to account for the right skewed distribution of health care costs and assure well-specified models.

Logistic models were used to estimate the associations of weight change category with risks of hospitalization and SNF stays (at least one episode) during the year following SOF Year 10 exam since only 522 women (25.1%) and 136 women (6.5%), respectively, had one or more hospital stays and one or more SNF stays.

Base models included Year 6 to Year 10 weight change category, Year 10 BMI category, age, time interval (months) between the Year 6 and Year 10 SOF exams, and study enrollment site as covariates. Only these covariates were forced into all multivariable models. Other covariates considered potential confounders or mediators of associations were added to the full multivariable models if their association with total health care costs (adjusted for the forced variables listed above) was significant at a p-value of <0.1. Since any association of weight loss with total health care costs might vary by category of BMI, we also tested for evidence of an interaction between weight change and BMI for prediction of costs.

## Results

### Baseline characteristics

Among the analytical cohort of 2,083 women (mean age 80.2 years, Table 1), 1,220 (58.6%) maintained their body weight, 594 (28.5%) experienced weight loss, and 269 (12.9%) experienced weight gain between the SOF Year 6 and Year 10 examinations (mean 4.5 years between exams. Some impairment of physical performance capability and functional status was common; the majority had a walk speed <1 m/sec, and 29% reported impairment in performing 2 or more IADLs. Twelve percent had moderate or severe depressive symptoms, and 24% had at least some cognitive impairment (mMMSE score 22 or less).

**Table 1:** Distribution of study population characteristics at SOF year 10 exam.

Individual Characteristics	All Participants (n = 2,083)	≥5% Weight Decrease (n = 594)	No Weight Change (n = 1,220)	≥5% Weight Increase (n = 269)
Total health care costs				
Median (IQR)	$1,897 (676 to 6,558)	$2,195 (705 to 8,631)	$1,757 644 to 5,514)	$1,982 (736 to 5,806)
Mean (SD)	$6,827 (12,671)	$8,536 (15,294)	$6,130 (11,148)	$6,217 (12,462)
Body mass index, kg/m^2^, N, (%)				
18.5 to 24.9	857 (41.1)	320 (53.9)	467 (38.3)	70 (26.0)
25.0 to 29.9	755 (36.2)	184 (31.0)	477 (39.1)	94 (34.9)
≥30	471 (22.6)	90 (15.2)	276 (22.6)	105 (39.0)
Age, years, mean (SD)	80.2 (4.4)	81.1 (4.8)	79.8 (4.1)	80.0 (4.4)
Education level, N (%)				
Less than high school	409 (19.6)	124 (20.9)	235 (19.3)	50 (18.6)
High school	836 (40.1)	252 (42.4)	475 (38.9)	109 (40.5)
<4 years of college	419 (20.1)	120 (20.2)	249 (20.4)	50 (18.6)
≥ 4years of college	419 (20.1)	98 (16.5)	261 (21.4)	60 (22.3)
Multimorbidity score (Elixhauser) N (%),				
0	357 (17.1)	85 (14.3)	234 (19.2)	38 (14.1)
1 or 2	963 (46.2)	232 (39.1)	602 (49.3)	129 (48.0)
3 or 4	505 (24.2)	168 (28.3)	271 (22.2)	66 (24.5)
5 or more	258 (12.4)	109 (18.4)	113 (9.3)	36 (13.4)
GDS score (0–15), N, (%)				
0 or 1	1,034 (49.6)	243 (40.9)	655 (53.7)	136 (50.6)
2 to 5	796 (38.2)	246 (41.4)	451 (37.0)	99 (36.8)
6 or more	253 (12.1)	105 (17.7)	114 (9.3)	34 (12.6)
mMMSE score (0–26), mean (SD)	23.8 (2.5)	23.5 (2.7)	23.9 (2.3)	23.6 (2.6)
Walk speed, m/sec, mean (SD)	0.89 (0.24)	0.83 (0.25)	0.93 (0.22)	0.88 (0.26)
Grip strength, lbs, mean (SD)	17.2 (4.1)	16.4 (4.0)	17.5 (4.0)	17.3 (4.1)
Number of impaired IADLs*, N (%)				
0	1,123 (54.0)	255 (43.1)	738 (60.6)	130 (48.5)
1	356 (17.1)	111 (18.8)	195 (16.0)	50 (18.7)
2 or more	599 (28.8)	226 (38.2)	285 (23.4)	88 (32.8)
Self-rated health,				
Good or excellent	1,644 (78.9)	431 (72.6)	1,006 (82.5)	207 (77.0)
Fair, poor, very poor	439 (21.1)	163 (27.4)	214 (17.5)	62 (23.0)

Abbreviations: IQR, interquartile range; SNF, skilled nursing facility; GDS, Geriatric Depression Scale; mMMSE, modified Mini Mental State Examination; IADL, instrumental activities of daily living

*Impaired IADLs defined as difficulty doing one or more of the five following tasks: walking 2–3 blocks, climbing up 10 steps, preparing meal, doing heavy housework and shopping; scored from 0 to 5

Compared to 2,425 women who were excluded on account of enrollment in Medicare Advantage and had BMI ≥ 18.5 kg/m^2^, there were statistically significant differences in age, walk speed, mMMSE score, grip strength, and number of impaired IADLs, but these differences were of very small magnitude (Table 2).

**Table 2:** Characteristics of 4605 women and according to enrollment and BMI status*.

Characteristic	All Participants (N = 4508)	FFS Enrollment		p-value
		Yes (N = 2083)	No (N = 2425)	
Body mass index, kg/m^2^, n (%)				0.148
18.5 to 24.9	1865 (40.5)	857 (41.1)	1008 (40.0)	
25 to 29.9	1662 (36.1)	755 (36.2)	907 (36.0)	
≥30	981 (21.3)	471 (22.6)	510 (20.2)	
Age, years, mean (SD)	80.3 (4.3)	80.2 (4.4)	80.5 (4.3)	0.018
Education (years), n (%)				0.12
Less than high school	852 (18.9)	409 (19.6)	443 (18.3)	
High school	1835 (40.7)	836 (40.1)	999 (41.2)	
Some college (<4 years)	959 (21.3)	419 (20.1)	540 (22.3)	
4 or more years of college	862 (19.1)	419 (20.1)	443 (18.3)	
GDS score (0–15), n (%)				0.15
0 or 1	2192 (48.7)	1034 (49.6)	1158 (48.0)	
2 to 5	1830 (39.8)	796 (38.2)	1034 (41.2)	
6 or more	538 (11.7)	253 (12.1)	285 (11.4)	
mMMSE score (0–26), mean (SD)	24.0 (2.4)	23.8 (2.5)	24.2 (2.4)	<0.001
Walk speed (m/s), mean (SD)	0.88 (0.23)	0.89 (0.24)	0.87 (0.22)	<0.001
Grip strength, lbs, mean (SD)	16.7 (4.2)	17.2 (4.1)	16.4 (4.3)	<0.001
IADL impairments (0–5), n (%)				0.017
0	2409 (53.6)	1123 (54.0)	1286 (53.2)	
1	846 (18.8)	356 (17.1)	490 (20.3)	
2 or more	1241 (27.6)	599 (28.8)	642 (26.6)	
Self-rated health status, n (%)				0.38
Good or excellent	3650 (79.4)	1644 (78.9)	2006 (79.8)	
Fair, poor, or very poor	947 (20.6)	439 (21.1)	508 (20.2)	

*Women with BMI <18.5 kg/m^2^ excluded

### Unadjusted health care utilization across weight change categories

Mean total health care costs for the year after the Year 10 SOF exam were $8,536 (SD 15,294 [2014 U.S. dollars]) for women with weight loss, $6,217 (SD 12,462) for women with weight gain, and $6,130 (SD 11,148) for women with weight maintenance (p-value for difference across means <0.01, Table 1). Larger proportions of women who lost weight went on to have one or more hospital stays (30.8%) and one or more SNF stays (9.1%) during the year after the SOF Year 10 visit, compared to women who gained weight (22.7% had hospital stays and 3.7% had SNF stays) or had stable weight (22.8% had hospital stays and 5.9% had SNF stays).

### Adjusted associations of weight change with health care costs and utilization (base model)

In the base model, adjusted for age, BMI at the Year 10 exam, study enrollment site, and the time interval over which weight change was tracked, weight loss before the Year 10 exam, was associated with a 35% increase of total health care costs during the year after the examination (cost ratio 1.35 [95% CI, 1.12 to 1.63); predicted incremental costs were $2,148 [95% CI, 745 to 3,552], 2014 U.S. dollars) (Table 3).

**Table 3:** Associations of weight change and BMI with total health care costs, outpatient costs, hospital stays, and SNF stays (base model without interaction terms)*^.

	Total Health Care Cost Ratio^ (95% CI)	Outpatient Care Cost Ratio (95% CI)	≥1 Hospital Stay, Odds Ratio (95% CI)	≥1 SNF Stay, Odds Ratio (95% CI)
Weight change				
None	Referent	Referent	Referent	Referent
≥5% Decrease	**1.35 (1.12, 1.63)**	**1.15 (1.00, 1.32)**	**1.42 (1.14, 1.76)**	**1.45 (1.03, 2.03)**
≥5% Increase	0.98 (0.76, 1.25)	1.08 (0.90, 1.29)	0.96 (0.67, 1.37)	0.58 (0.28, 1.20)
Body mass index, kg/m^2^				
18.5 to 24.9	Referent	Referent	Referent	Referent
25.0 to 29.9	1.14 (0.95, 1.38)	1.14 (0.99, 1.30)	1.14 (0.92, 1.42)	1.35 (0.87, 2.09)
≥30	1.10 (0.88, 1.36)	1.04 (0.88, 1.22)	1.19 (0.90, 1.58)	1.34 (0.82, 2.19)
Age (per 5 year increase)	**1.20 (1.09, 1.31)**	0.95 (0.89, 1.02)	**1.37 (1.22, 1.54)**	**1.67 (1.36, 1.98)**
Constant†	$5,584 (2,973 to 10,485)	$1,821 (1,138 to 2,913)	N/A	N/A

*Significant cost ratios and odds ratios at p-value <0.05 are in **bold**

^Also adjusted for study enrollment site and time interval (between SOF Year 6 and Year 10 exams) over which body weight change was recorded

^†^Constant values represent mean predicted costs for individuals at mean age (80.2 years) and categorical variables at references levels (no weight change and normal current BMI)

Similarly, weight loss compared with weight maintenance was associated with 15% higher outpatient care costs (cost ratio 1.15 [95% CI, 1.00 to 1.32]; predicted incremental costs $329 [95% CI, −1 to 660]), a higher incident odds of one or more hospital stays (OR 1.42 [95% CI, 1.14 to 1.76]) and one or more SNF stays (OR 1.45 [95% CI, 1.03 to 2.03]) (Table 3). Neither weight gain before the Year 10 exam nor being obese or overweight BMI at the SOF Year 10 visit were associated with subsequent total health care costs, outpatient costs, hospital stays, or SNF stays (Table 3).

The associations of weight loss with total health care costs, outpatient costs, hospital stays, and SNF stays did not vary by BMI category (p-value for all interaction terms between weight change and BMI categories >0.10 with all four dependent variables). Considering a model with main effects and interactions, weight loss compared to weight maintenance had a cost ratio or 1.38 (95% CI 1.06 to 1.79) and with associated interaction terms of 0.98 (95% CI 0.66 to 1.46 for weight loss among those who were overweight, and 0.95 (95% CI 0.57 to 1.56) for weight loss among those were obese, thus indicating minimal variation of this association by BMI category.

### Full multi-variable adjusted associations of weight change with health care costs and utilization

Multi-morbidity, impaired IADLs, depressive symptoms, walk speed, grip strength, and self-rated health were each associated with total health care costs, adjusted for age, weight change category, BMI, study enrollment site, and time interval over which weight change was recorded, (Table 4) whereas educational status and MMSE score were not.

**Table 4:** Associations of additional predictor covariates with total health care costs for 1 year after SOF year 10 exam, adjusted only for base model covariates*†.

Individual Predictors	Cost Ratio (95% CI)
Education level	
Less than high school	Referent
High school	1.14 (0.91, 1.42)
Some college (<4 years)	1.08 (0.83, 1.40)
4 or more years of college	1.06 (0.82, 1.35)
Elixhauser multimorbidity score (0–28)	
0	Referent
1 or 2	**1.70 (1.37, 2.11)**
3 or 4	**2.57 (2.01, 3.28)**
5 or more	**4.28 (3.19, 5.72)**
GDS score (0–15)	
0 or 1	Referent
2 to 5	**1.58 (1.33, 1.87)**
6 or more	**2.04 (1.59, 2.63)**
mMMSE score (for 1 SD decrease)	1.06 (0.97, 1.15)
Walk speed (for 1 SD decrease)	**1.30 (1.19, 1.43)**
Grip strength (for 1 SD decrease)	**1.12 (1.03, 1.22)**
IADL impairment	
0	Referent
1	**1.56 (1.25, 1.95)**
2 or more	**2.36 (1.94, 2.87)**
Self-rated health	
Good or excellent	**Referent**
Fair, poor, or very poor	**1.59 (1.31, 1.93)**

*Adjusted for age, BMI category at visit 6, change in weight between visits 4 and 6, time between visits 4 and 6, and study enrollment site.

^†^ Significant odds ratios at p-value <0.05 are in **bold**

After consideration of multiple potential confounders and mediators, including multi- morbidity burden and impairment in IADL, weight loss no longer was associated with cost and health care utilization outcomes (Table 5). However, multi-morbidity, impaired IADLs, and depressive symptoms each were independent predictors of total health care and outpatient costs, and hospital stays. Multi-morbidity and impaired IADLs also were independently associated with SNF stays (Table 5). Walk speed, grip strength, and self-rated health were not independently associated with total health care costs after full multivariable adjustment.

**Table 5:** Full multivariable adjusted associations of weight change and BMI with total health care costs, outpatient costs, hospital stays, and SNF stays*^.

Predictor	Total Health Care Cost Ratio (95% CI)	Outpatient Cost Ratio (95% CI)	≥1 Acute Hospital Stay, Odds Ratio (95% CI)	≥1 SNF Stay, Odds Ratio (95% CI)
Weight change				
None	Referent	Referent	Referent	Referent
≥5% Decrease	1.05 (0.87, 1.26)	0.96 (0.84. 1.09)	1.12 (0.90, 1.39)	1.10 (0.77, 1.59)
≥5% Increase	0.85 (0.67, 1.08)	1.01 (0.85, 1.19)	0.87 (0.61, 1.25)	0.53 (0.26, 1.10)
Body mass index, kg/m^2^				
18.5 to 24.9	Referent	Referent	Referent	Referent
25.0 to 29.9	1.03 (0.86, 1.23)	1.04 (0.92, 1.18)	1.05 (0.85, 1.30)	1.18 (0.77, 1.80)
≥30	0.97 (0.78, 1.20)	0.95 (0.82, 1.10)	0.97 (0.72, 1.31)	1.05 (0.61, 1.80)
Age (per 5 year increase)	1.09 (0.99, 1.19)	**0.90 (0.84. 0.96)**	1.19 (1.06, 1.35)	1.39 (1.13, 1.71)
Multimorbidity score (Elixhauser, 0–28)				
0	Referent	Referent	Referent	Referent
1 or 2	**1.54 (1.24, 1.91)**	**1.44 (1.23, 1.68)**	**1.49 (1.09, 2.03)**	0.84 (0.49, 1.45)
3 or 4	**2.14 (1.68, 2.74)**	**1.92 (1.62, 2.29)**	**2.12 (1.45, 3.11)**	1.25 (0.65, 2.39)
5 or more	**3.28 (2.44, 4.41)**	**2.60 (2.11. 3.21)**	**3.26 (2.17, 4.90)**	**2.08 (1.04, 4.15)**
Impaired IADLs				
0	Referent	Referent	Referent	Referent
1	**1.45 (1.17, 1.80)**	**1.17 (1.01, 1.37)**	**1.70 (1.26, 2.30)**	1.14 (0.60, 2.18)
2	**1.66 (1.35, 2.03)**	**1.23 (1.07, 1.42)**	**1.99 (1.56, 2.54)**	**2.13 (1.27, 3.58)**
GDS score (0–15)				
0 or 1	Referent	Referent	Referent	Referent
2 to 5	**1.30 (1.09, 1.54)**	**1.26 (1.11, 1.42)**	1.14 (0.91, 1.43)	**1.85 (1.20, 2.84)**
6 or more	**1.39 (1.07, 1.81)**	**1.24 (1.03, 1.49)**	**1.44 (1.00, 2.08)**	**1.78 (1.04, 3.06)**

*All models also adjusted for study enrollment site and time interval (between SOF Year 6 and Year 10 exams) over which body weight change was recorded

^ Significant odds ratios at p-value <0.05 are in **bold**

## Discussion

In this population of community-dwelling women late in life, recent weight loss was associated with greater health care burden, characterized by higher total health care costs, hospital stays, SNF stays, and outpatient costs. The association of weight loss with higher health care costs was consistent across BMI category. However, associations of weight loss with greater health care costs and utilization were largely attributable to greater multi-morbidity burden and disability among women with weight loss. By comparison, recent weight gain was not associated with health care utilization.

Since weight loss is a marker of prevalent and incident multi-morbidity in older individuals, [1] it is not surprising that its association with total health care costs may be explained by greater multi-morbidity burden among those adults with recent weight loss, regardless of current BMI. Importantly, our results also indicate that weight loss may be associated with higher health care costs among the very old in part because weight loss is associated with functional impairment, which in turn is associated with health care costs and utilization even after adjustment for multimorbidity. While our findings do not in any way show that weight loss causes higher health care utilization, it is possible that the association of weight loss with higher health care utilization may be partially mediated by functional impairment,. Weight loss may result in reduction of muscular strength and functional impairment even among obese individuals, because weight loss typically entails loss of both lean and fat body mass. [19] The detrimental effects of lean mass loss may be pronounced among very older adults who already have experienced significant age-related loss of muscle mass, [20] and may be a cause of functional decline [21] and incident frailty. [22] Hence, weight loss might be a marker of those with changing needs requiring additional assistance, adaptive devices to live independently, and health care in more expensive care settings (hospital or skilled nursing facilities vs community care). Further investigations are needed to establish if this hypothesized causal pathway is true.

Our results are in contrast to those of Wilkens and colleagues, [5] who noted that weight loss (based on differences in self-reported weight) among respondents to the Medicare Beneficiary Current Survey (without adjustment for multi-morbidity or disability) was not associated with health care costs for those age 75 years or older (98.4% of our study population was age 75 and older). However, the bias introduced by self-reported weight conceivably could lead to different study findings than investigations that use actually measured height and weight. [23]

Weight loss is often recommended for people with medical conditions exacerbated by excess weight, such as diabetes mellitus, [24] coronary artery disease, [25] and osteoarthritis of weight-bearing joints, [26], and in obese very old individuals weight loss may still be important for management of specific comorbid conditions. However, our study suggests that intentional weight loss in obese very old individuals should be accompanied by appropriate exercise and nutritional interventions to reduce or prevent the loss of lean mass and muscle strength that can accompany weight loss, and to preserve functional status. [27, 28]

### Study limitations and strengths

There are several limitations of our analyses. First, we could not distinguish between intentional and unintentional weight loss. However, this is mitigated by the facts that sustained weight loss in the elderly is rarely intentional and is difficult, and individuals with multi-morbidity or other causes of weight loss may be more successful when intentionally trying to lose weight. Second, our cohort was established in the late 1980s and cost data were primarily from the late 1990s, but there is no apparent reason why our findings would not be applicable today. Fourth, we were underpowered to examine robustly the association of weight changes and health care costs in among very thin women (BMI <18.5 kg/m^2^) or those with severe obesity (BMI ≥35 kg/m^2^). Similarly, we did not have adequate power to assess the association between lesser degrees of weight loss (2.5% to 5% of body weight [29]) and health care utilization.

Our study also has several important strengths. Individual characteristics are carefully assessed and measured in the SOF cohort, and by merging these data to Medicare claims, analyses using these data are uniquely positioned to examine the association of measured individual patient characteristics with total health care costs. Body weight and height were measured, rather than self-reported. Ours is the only study of the associations of measured weight change and BMI with health care costs to specifically focus on individuals age 75 and older, and to include adjustment for both multimorbidity and functional impairment.

In conclusion, weight loss among older women is not associated with higher total health care costs, outpatient costs, hospital utilization, and SNF utilization, after accounting for multi-morbidity and functional impairment.
